# Underestimated Amoebic Appendicitis among HIV-1-Infected Individuals in Japan

**DOI:** 10.1128/JCM.01757-16

**Published:** 2016-12-28

**Authors:** Taiichiro Kobayashi, Koji Watanabe, Hideaki Yano, Yukinori Murata, Toru Igari, Kumiko Nakada-Tsukui, Kenji Yagita, Tomoyoshi Nozaki, Mitsuo Kaku, Kunihisa Tsukada, Hiroyuki Gatanaga, Yoshimi Kikuchi, Shinichi Oka

**Affiliations:** a AIDS Clinical Center, National Center for Global Health and Medicine, Shinjuku, Tokyo, Japan; b Department of Infection Control and Laboratory Diagnostics, Internal Medicine, Tohoku University Graduate School of Medicine, Sendai, Miyagi, Japan; c Department of Parasitology, National Institute of Infectious Diseases, Shinjuku, Tokyo, Japan; d Department of Medicine, University of Virginia, Charlottesville, Virginia, USA; e Department of Surgery, National Center for Global Health and Medicine, Shinjuku, Tokyo, Japan; f Pathology Division of Clinical Laboratory, National Center for Global Health and Medicine, Shinjuku, Tokyo, Japan; g Graduate School of Life and Environmental Sciences, University of Tsukuba, Tsukuba, Ibaraki, Japan; University of Texas Medical Branch

**Keywords:** Entamoeba histolytica, Japan, PCR, S^TGA^-D locus, appendicitis

## Abstract

Entamoeba histolytica is not a common causative agent of acute appendicitis. However, amoebic appendicitis can sometimes be severe and life threatening, mainly due to a lack of awareness. Also, its frequency, clinical features, and pathogenesis remain unclear. The study subjects were HIV-1-infected individuals who presented with acute appendicitis and later underwent appendectomy at our hospital between 1996 and 2014. Formalin-fixed paraffin-embedded preserved appendix specimens were reexamined by periodic acid-Schiff (PAS) staining and PCR to identify undiagnosed amoebic appendicitis. Appendectomies were performed in 57 patients with acute appendicitis. The seroprevalence of E. histolytica was 33% (14/43) from the available stored sera. Based on the medical records, only 3 cases were clinically diagnosed as amoebic appendicitis, including 2 diagnosed at the time of appendectomy and 1 case diagnosed by rereview of the appendix after the development of postoperative complications. Retrospective analyses using PAS staining and PCR identified 3 and 3 more cases, respectively. Thus, E. histolytica infection was confirmed in 9 cases (15.8%) in the present study. Apart from a significantly higher leukocyte count in E. histolytica-positive patients than in negative patients (median, 13,760 versus 10,385 cells/μl, respectively, *P* = 0.02), there were no other differences in the clinical features of the PCR-positive and -negative groups. In conclusion, E. histolytica infection was confirmed in 9 (15.8%) of the appendicitis cases. However, only 3, including one diagnosed after intestinal perforation, were diagnosed before the present analyses. These results strongly suggest there is frequently a failure to detect trophozoites in routine examination, resulting in an underestimation of the incidence of amoebic appendicitis.

## INTRODUCTION

Appendicitis is one of the most common causes of acute surgical abdomen. The reported lifetime prevalence is 5 to 10% (1). Although the exact pathogenic cause of appendicitis is still unclear, internal obstruction (e.g., by a fecalith, lymphoid hyperplasia, or tumor) and direct invasion of microorganisms are some of the suggested etiologies of appendicitis (2). Although the majority of causative microorganisms are bacteria, cases of appendicitis caused by Entamoeba histolytica (amoebic appendicitis) have been reported (3, 4).

E. histolytica is a protozoa endemic throughout the world, especially in developing countries (5). More than 50 million people are affected by E. histolytica, and amoebiasis is the second leading cause of mortality due to parasitic infestation, with over 100,000 deaths annually (6). Amoebiasis is a food- or waterborne disease in developing countries. On the other hand, in the developed countries of East Asia and Oceana, amoebiasis is considered a sexually transmitted infection (STI) and a waterborne (nonsexually transmitted) infection, which commonly affects travelers. HIV-1-infected men who have sex with men (MSM) are recognized as risk factor for sexually transmitted amoebiasis in these countries (7–9). Importantly, the number of cases of amoebiasis in Japan is increasing annually, mainly as STIs (10).

Two review articles reported the clinical features of amoebic appendicitis (11, 12). The oversight of E. histolytica infection sometimes results in serious complications after appendectomy. It was reported that amoebic appendicitis presents a high rate of perforated appendicitis at surgery (26.1%) and a high rate of postoperative complications (25.4%) (12, 13). Also, several case reports indicated that the diagnosis of E. histolytica infection of the appendix was established at autopsy (14, 15). On the other hand, the prevalence of E. histolytica infection among appendicitis cases has never been evaluated rigorously. Moreover, amoebic appendicitis might have been underestimated in previous studies because the diagnosis of E. histolytica in resected tissue generally relies only on hematoxylin and eosin (H&E) staining, which has a relatively low sensitivity for detecting E. histolytica compared with those of periodic acid-Schiff (PAS) staining and PCR (16).

In the present study, formalin-fixed paraffin-embedded (FFPE) preserved appendix samples of HIV-1-infected appendicitis patients were rigorously examined by PAS staining and PCR to determine the exact prevalence, clinical features and pathogenesis of amoebic appendicitis.

## RESULTS

### Study population and review of medical records.

A total of 57 HIV-1-infected individuals presented with acute appendicitis and underwent appendectomy during the study period. A review of the medical records indicated the diagnosis of acute appendicitis was caused by Entamoeba in only 3 cases (5.3%). In 2 of these 3 cases, the primary physicians suspected amoebic infection based on the clinical characteristics, such as Japanese HIV-1-infected patients, MSM, and past medical history of amoebiasis, although these patients showed only typical clinical features of acute appendicitis. In these two patients, Entamoeba trophozoites were detected by direct microscopic examination of ascites fluid at surgery and confirmed by histopathological examination of the resected appendices (H&E and PAS stains). Both patients received full courses of metronidazole therapy after the appendectomies and the postoperative periods were uneventful. In the third patient, amoebiasis was not suspected clinically at surgery and Entamoeba was not identified on the initial histopathological assessment with H&E staining. The patient developed intestinal perforation, an intraabdominal abscess, and an enterocutaneous fistula on postoperative day (POD) 14, and the presence of Entamoeba was confirmed in the resected appendix on a repeated histopathological examination (H&E and PAS stains). The patient subsequently underwent ileocecal resection at POD 130, though treatment with metronidazole was provided for 2 weeks (from POD 34 to POD 47), and Entamoeba was not detected in the resected ileocecal sample.

### Identification of Entamoeba by PAS stain.

**FIG 1:**
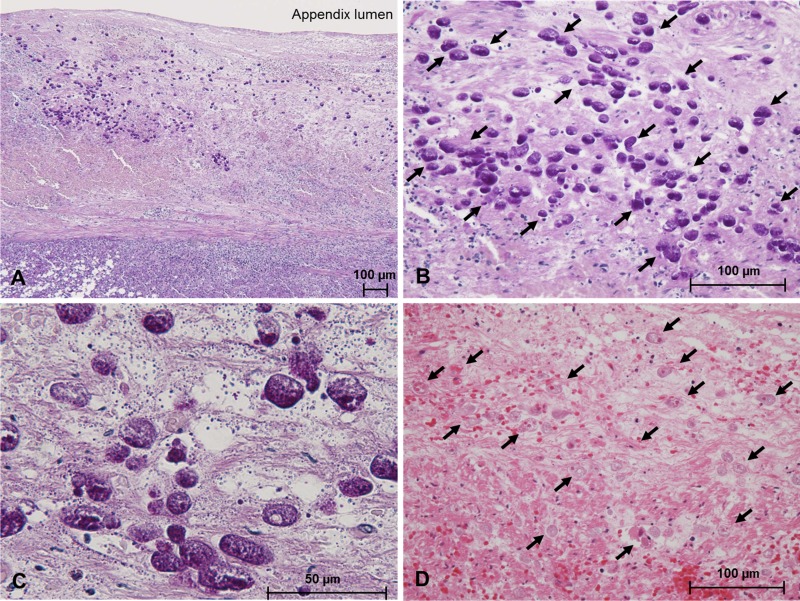
Representative histopathological findings of an Entamoeba-positive appendix (case AA19). The cytoplasm of the invading trophozoites of Entamoeba (arrows in B) is clearly stained with periodic acid-Schiff (PAS) stain (magnification, ×100 [A]; ×400 [B]; ×1,000 [C]). Both trophozoites (arrows) and necrotic tissue are stained with eosin using a hematoxylin and eosin stain (D; magnification, ×400).

Next, we performed additional PAS staining using all preserved FFPE samples of appendices to detect Entamoeba-infected cases undiagnosed by routine H&E staining. The PAS staining identified three new cases of amoebiasis. While Entamoeba was not detected by H&E staining in these 3 cases at the time of surgery based on the medical records, we identified the protozoon on H&E-stained slides at the same microscope field where it was detected by PAS staining. Entamoeba invasion of the submucosal tissue was clearly observed in all these three cases (representative pictures of histopathology are shown in Fig. 1).

### Identification of E. histolytica DNA in FFPE specimens by PCR.

**FIG 2:**
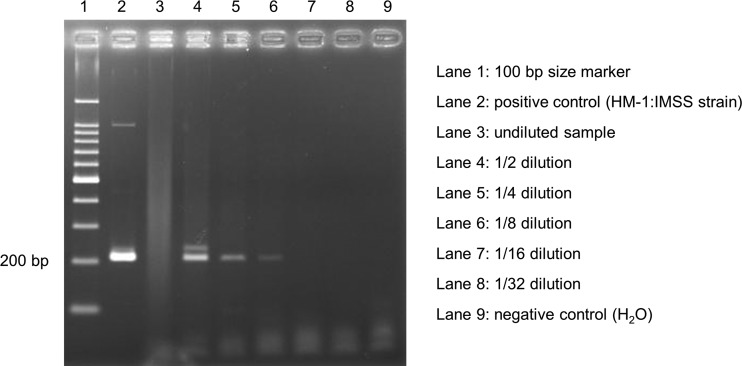
Results of DNA amplification using Entamoeba histolytica-specific S^TGA^-D targeted primers in a representative sample from an E. histolytica-positive patient (case AA38). Sequencing was performed using the amplicon shown in lane 5, which showed a clear and single band, and genotype was determined as 15SD.

For a more sensitive detection of E. histolytica and to distinguish E. histolytica from other Entamoeba species, we applied PCR to the FFPE samples using E. histolytica-specific primers. We compared the sensitivities and specificities of 24 candidate primer sets using the sample from the patient who developed intestinal perforation (case AA56) as a positive control of amoebic appendicitis. The protocol described below confirmed that the primer set targeting the S^TGA^-D locus in the tRNA region was optimal for detection of E. histolytica DNA in the FFPE samples of human appendices. The primers were S^TGA^D-H5, 5′-AAATCCTGCCACTGTCGTAA-3′, and S^TGA^D-H3, 5′-AATCCCCGTTGAAGAGTTCT-3′, and the optimal annealing temperature was 60°C (17, 18). All six samples in which Entamoeba was histopathologically identified were E. histolytica-positive by PCR using S^TGA^-D targeted primers (a representative result of PCR is shown in Fig. 2). Next, PCR using the above primers was applied in a blinded fashion using other samples in which Entamoeba was not detected by histopathological examination. Among the 51 histopathologically negative samples for Entamoeba, 3 FFPE samples were positive by PCR. These 3 cases did not include any of the above-described 6 cases. Although the presence of E. histolytica was identified by PCR in these 3 cases, Entamoeba could not be detected histopathologically in the appendix tissues. Interestingly, PCR using undiluted template showed a negative result in 6 of 9 PCR-positive cases, probably due to inhibition of the amplification as shown in Fig. 2 (lane 3).

Sequence analysis of all nine E. histolytica positive samples was performed to determine the genotypes from short tandem repeat (STR) types in the S^TGA^-D locus. The STR types were diverse and showed four different genotypes. Two genotypes (12SD and 15SD) were commonly seen in previous reports from Japan, whereas the other two (8SD and 16SD) were rarely reported genotypes (17, 19). Two different genotypes were identified in one sample (case AA57), indicating a mixed infection with two different genotypes of E. histolytica in this patient (Table 1).

**TABLE 1:** Diagnostic evaluation and sequence results in S^TGA^-D locus in 9 patients with PCR-confirmed amoebic appendicitis

Case no.	Pathological finding on medical records	Histopathological re-examination		E. histolytica-specific PCR	Sequence type
		PAS stain	H&E stain		
AA19	Nonamoebic	Positive	Positive	Positive	8SD
AA25	Nonamoebic	Positive	Positive	Positive	12SD
AA33	Amoebic	Positive	Positive	Positive	12SD
AA38	Nonamoebic	Positive	Positive	Positive	15SD
AA42	Nonamoebic	Negative	Negative	Positive	16SD
AA44	Nonamoebic	Negative	Negative	Positive	15SD
AA45	Nonamoebic	Negative	Negative	Positive	15SD
AA56	Amoebic	Positive	Positive	Positive	12SD
AA57^*a*^	Amoebic	Positive	Positive	Positive	12SD/15SD

a Entamoeba infection was missed on initial histopathological examination with H&E staining at appendectomy but was identified on rereview of the appendix specimen with PAS and H&E staining at 14 days after surgery when the patient developed intestinal perforation.

In summary, E. histolytica infection was confirmed by PCR in 9 of 57 (15.8%) HIV-1-infected patients with appendicitis who underwent appendectomy.

### Characteristics of the clinical course.

To identify the clinical features of amoebic appendicitis, we compared the patient characteristics and clinical presentations at surgery between amoebic and nonamoebic appendicitis patients (Table 2). Most of the appendicitis patients were Japanese and male, and 82% were MSM. CD4 counts were less than 200 cells/μl in 11 patients (19%). One patient was concurrently treated for tuberculous meningitis and two patients received secondary prophylaxis for Mycobacterium avium complex lymphadenitis at appendectomy. Twelve percent had histories of amoebiasis prior to developing appendicitis, and seropositivity from available stored sera for E. histolytica antibodies was 33% (14/43), although these values were not significantly different between amoebic and nonamoebic appendicitis cases. These data suggested that HIV-1-infected individuals in Japan who developed appendicitis were highly affected by E. histolytica. Even among the patients with amoebic appendicitis, only a few showed symptoms of colitis, such as diarrhea (1/9 [11%]) and dysentery (1/9 [11%]). The median time from the onset of abdominal pain to surgery was 3 days (range, 1 to 15 days) and did not differ between the two groups. Leukocyte counts at surgery were higher in patients with amoebic appendicitis than in those without (13,760 versus 10,385 cells/μl, *P* = 0.02), and there were significantly more cases with leukocyte counts >10,000 cells/μl among amoebic appendicitis cases than among nonamoebic cases (*P* < 0.01), whereas no differences were seen in other clinical, imaging, and laboratory findings between the two groups. It is not surprising that none of the patients was diagnosed with amoebic appendicitis before surgery, since these clinical features are nonspecific. Attending physicians provided metronidazole treatment at 500 mg *ter in die* (TID) for 15 days or 750 mg TID for 10 days to 5 patients in the amoebic appendicitis group and to 3 patients in the nonamoebic appendicitis group. Only one patient with amoebic appendicitis who did not receive metronidazole immediately after appendectomy developed an enterocutaneous fistula and an intraabdominal abscess (as described above), whereas the other 8 amoebic appendicitis cases, including the 3 cases who were not treated with metronidazole, did not develop postoperative complications or invasive amoebiasis thereafter. No fatalities were recorded in patients of the amoebic appendicitis group.

**TABLE 2:** Characteristics of participating patients

Characteristic	Cases^*a*^			*P* value^*b*^
	All (*n* = 57)	Amoebic appendicitis (*n* = 9)	Nonamoebic appendicitis (*n* = 48)	
Age (years)	34 (22–70)	32 (26–46)	35 (22–70)	0.53
Male sex	55 (96)	9 (100)	46 (96)	0.71
MSM	47 (82)	8 (89)	39 (81)	0.50
Japanese nationality	53 (93)	7 (78)	46 (96)	0.11
History of amoebiasis	7 (12)	2 (22)	5 (10)	0.30
Antiretroviral therapy	38 (67)	5 (56)	33 (69)	0.34
CD4 count (cells/μl)	400 (56–1,443)	497 (159–880)	399 (56–1,443)	0.24
HIV-RNA (log copies/ml)	UD^*d*^ (UD–5.4)	2.4 (UD–5.1)	UD (UD–5.4)	0.45
Disease duration (days)	3 (1–15)	2 (1–15)	3 (1–13)	0.67
Fever	19 (33)	4 (44)	15 (31)	0.34
Abdominal pain	57 (100)	9 (100)	48 (100)	na
Diarrhea	5 (9)	1 (11)	4 (8)	0.59
Dysentery	1 (2)	1 (11)	0 (0)	0.16
Leukocyte count (cells/μl)	11,160 (3,590–26,060)	13,760 (10,100–18,200)	10,385 (3,590–26,060)	0.02
CRP^*c*^ (mg/dl)	2.79 (0.01–37.7)	8.43 (1.13–15.4)	2.46 (0.01–37.7)	0.21
E. histolytica antibody positive	14/43 (33)	4/9 (44)	10/34 (29)	0.31
Fecalith	18/56 (32)	3/9 (33)	15/47 (32)	0.61
Peritonitis	29 (51)	4 (44)	25 (52)	0.47

a Data are presented as *n* (%) of patients or median (range).

b *P* values represent comparisons of continuous and categorical variables by Mann–Whitney *U* and Fisher's exact tests, respectively.

c CRP, C-reactive protein.

d UD, undetectable.

## DISCUSSION

The diagnosis of amoebic appendicitis is very difficult in clinical settings because the clinical picture is similar to that of nonamoebic appendicitis. One previous systematic review showed that preoperative diagnosis was established in only 3.0% of the patients (12). On the other hand, there are no epidemiological studies that used highly sensitive methods to detect E. histolytica. In most reports, the diagnosis of amoebic appendicitis relied on clinical findings, direct microscopic examination of stool, and histopathological assessment by H&E staining (11–15). Therefore, we hypothesized that the prevalence of amoebic appendicitis is currently underestimated. In the present study, 9 of 57 (15.8%) HIV-1-infected patients who underwent appendectomy were confirmed to have amoebic appendicitis by PCR, whereas only 2 amoebic appendicitis patients were diagnosed at appendectomy. Moreover, none of the cases were diagnosed preoperatively as amoebic appendicitis. On the other hand, 75.0% (3/4) of our amoebic appendicitis patients who were not prescribed metronidazole at surgery did not develop postoperative complications, indicating that perioperative treatment with metronidazole is not necessarily required for preventing postoperative complications. Therefore, a number of undiagnosed amoebic appendicitis cases are expected under endemic conditions of E. histolytica. Most of the undiagnosed cases with E. histolytica infection might fully recover after appendectomy without metronidazole treatment. However, in some cases, oversight of E. histolytica infection at surgery leads to life-threatening complications, as one patient in this study suffered intestinal perforation and intraabdominal abscess and enterocutaneous fistula formations, and later required ileocecal resection. Although we could not identify the factors that predict postoperative complications due to the small sample size, clinicians should make more of an effort to rule out E. histolytica infection in patients presenting with acute appendicitis under endemic settings of E. histolytica.

Our results confirmed that PCR using FFPE samples is more sensitive than histopathology using H&E and PAS staining for the diagnosis of amoebic appendicitis. However, PCR is still technically complex for routine examinations, especially in resource-limited settings. Even in developed countries, clinicians have to first suspect E. histolytica infection based on clinical findings before they request PCR as a nonroutine test for a resected appendix. For this reason, we tried to define the clinical features of amoebic appendicitis. However, we found that most clinical symptoms and laboratory findings, other than higher leukocyte counts at the onset, were not different between amoebic and nonamoebic appendicitis (Table 2). Thus, we conclude that it is often difficult to differentiate amoebic appendicitis from a nonamoebic one based on the clinical picture only during the acute phase of appendicitis.

The pathogenesis of amoebic appendicitis, such as the incubation period of parasite infection, is still unclear, although a previous meta-analysis indicated that subclinical chronic infection of E. histolytica continues for months or years before the development of amoebic appendicitis (12). Also, in this study, preliminary results of repeated cytokine analyses using stocked samples from one patient (case AA42) showed high serum levels of interleukin-4 (IL-4), a marker of asymptomatic E. histolytica infection (20), at 3.5 months before the onset of acute appendicitis, and the level decreased immediately after treatment of amoebic appendicitis (data not shown). Unfortunately, no control data were available for serum IL-4 levels in the HIV-1-infected individual who did not have E. histolytica infection, and thus the significance of this finding remains to be confirmed in future studies. Further research of the pathogenesis of amoebic appendicitis is needed. Such studies are necessary for reducing amoeba-related morbidity and mortality in acute appendicitis by detecting subclinical chronic E. histolytica infection and providing preemptive treatment.

This research has some limitations that should be considered. First, this was a single-center retrospective study involving a small sample size. The exact prevalence and clinicopathological assessment of amoebic appendicitis will be explored in future studies. Moreover, retrospective study of long-term preserved FFPE samples can lead to false negative PCR results. In fact, the PCR positivity rate of histologically negative FFPE samples fixed for more than 5 years was 0% (0/36), whereas the positivity rate in samples fixed fewer than 5 years ago was 20% (3/15) (*P* = 0.02) (see Fig. S1 in the supplemental material). Our results might show that at least 15.8% of the cases are amoebic appendicitis. Second, we selected HIV-1-infected individuals for the present analyses because they are affected by E. histolytica to a greater extent than non-HIV ones in our country (10, 21). It is expected that the rate of amoebiasis is higher among immunocompromised HIV-1-infected individuals than among uninfected ones (22–24), although it was reported that clinical presentations are not influenced by the presence of HIV-1 (19, 25, 26).

In conclusion, our results suggest that the true prevalence of amoebic appendicitis is underestimated under endemic situations of E. histolytica. Furthermore, our results emphasize the need for large epidemiological prospective studies using PCR under endemic settings of E. histolytica to determine the risk factors, clinical features, best diagnostic procedure, and treatment strategy for amoebic appendicitis.

## MATERIALS AND METHODS

### Study design and population.

This was a single-center retrospective cohort study. Our medical center is a teaching hospital with 800 beds and the largest referral center for HIV/AIDS in Japan. At the Department of Pathology, clinical pathologists examine the tissue slides and make pathological diagnoses. All collected FFPE samples are preserved.

We reviewed the medical records of all 3,827 HIV-1-infected patients who received medical care at our center between 1996 and 2014, and we selected all cases diagnosed as acute appendicitis who underwent appendectomy. The diagnosis of acute appendicitis was confirmed by pathological evidence of an inflamed appendix wall. The clinical characteristics and clinical course were retrieved from the medical records.

In this study, the diagnosis of amoebic appendicitis was established in three separate steps. In the first step, the diagnosis was established when the patient was managed clinically at the hospital between 1996 and 2014. To identify the diagnosis, we reviewed the medical records of all patients with a final diagnosis of acute appendicitis and selected patients who were diagnosed as amoebic appendicitis during the perioperative period. Infection with Entamoeba was confirmed by microbiological and histopathological examinations at the time of the original diagnosis. In the second step, the diagnosis was established by reexamining histopathologically the slides of all preserved tissue samples by PAS staining to detect Entamoeba in the appendix wall. At the time of the study, all tissue samples had already been examined by H&E staining. In the third step, the diagnosis was established by performing E. histolytica-specific PCR using the preserved FFPE samples from all cases.

This study was approved by the ethics committee of the National Center for Global Health and Medicine (NCGM-G-001458-01) and conducted in accordance with the Declaration of Helsinki.

### E. histolytica-specific PCR and sequencing using FFPE samples of appendices.

The QIAamp DNA FFPE tissue kit (Qiagen, Hilden, Germany) was used to remove paraffin and extract DNA from the FFPE samples according to the instructions provided by the manufacturer. We prepared 25 sections (10-μm thick) from each FFPE appendix specimen (total thickness per case, 250 μm). The DNA was finally eluted with 250 μl of nuclease-free water (Qiagen). The extracted DNA was purified and concentrated by the NucleoSpin gDNA cleanup XS kit (Macherey-Nagel, Düren, Germany), because the extraction liquid was expected to contain a large amount of impurities and a small amount of E. histolytica DNA. The purified DNA samples were finally concentrated into 10 μl of buffer BE. Then, DNA templates for PCR were prepared as 2-fold serial dilutions from 1× to 32× (6 templates from 1 specimen) with nuclease-free water.

For E. histolytica-specific PCR, we selected candidate primers targeting a relatively short region (less than 300 bp) based on previous reports using human clinical samples (17, 18, 27–35). This approach was taken because formalin fixation and long-term preservation in paraffin can cause DNA to degenerate into small fragments. Also, we designed several new primers using Primer-BLAST (36) to target small subunit rRNA regions. PCR was performed with 2 μl of template DNA in a 20 μl reaction mixture that included 0.4 μl Tks Gflex DNA polymerase (TaKaRa Bio, Shiga, Japan), 2× Gflex PCR buffer, and 6 pmol of each primer. The PCR conditions were as follows. After a 2-min hold at 98°C, 45 cycles (98°C for 10 s, 50 to 62°C for 30 s, and 68°C for 15 s) were followed by a final extension at 68°C for 7 min. PCR using a nontemplate control was simultaneously performed. All positive PCR products were cloned into the pCR-Blunt II-TOPO (Invitrogen, Carlsbad, CA) plasmid vector, transformed into One Shot TOP10 chemically competent Escherichia coli cells (Invitrogen), spread on imMedia growth medium agar plates containing ampicillin (Invitrogen), and incubated overnight at 37°C. Ten randomly selected white colonies were subjected to colony PCR and sequenced. Direct DNA sequencing was performed by the BigDye Terminator v3.1 cycle sequencing kit (Applied Biosystems, Foster, CA) according to the instructions provided by the manufacturer using a model 3730 automated DNA analyzer (Applied Biosystems). The fragments containing the target gene were analyzed using GENETIX version 11 software (GENETIX, Tokyo, Japan).

### Statistical analysis.

The clinical characteristics at appendectomy were compared using Fisher's exact and Mann–Whitney *U* tests for categorical and continuous variables, respectively. Statistical significance was defined as a two-sided *P* value of < 0.05. All statistical analyses were performed with the Statistical Package for the Social Sciences (SPSS) version 23.0 (IBM, Chicago, IL).

## Supplementary Material

Supplemental material
