# Supplementary material for: Underestimated Amoebic Appendicitis among HIV-1-Infected Individuals in Japan
Source: J Clin Microbiol. 2016 Dec 28;55(1):313–20. doi: 10.1128/JCM.01757-16 (PMC5228245; doi:10.1128/JCM.01757-16)
Supplement: Supplemental material [file supp_55_1_313__index.html]

Supplemental material 

# Underestimated Amoebic Appendicitis among HIV-1-Infected Individuals in Japan

## Supplemental material

- Supplemental file 1 -

  Fig. S1 (Results of PCR using histologically negative samples)

  PDF, 22K
