# Supplementary material for: Underestimated Amoebic Appendicitis among HIV-1-Infected Individuals in Japan
Source: J Clin Microbiol. 2016 Dec 28;55(1):313–20. doi: 10.1128/JCM.01757-16 (PMC5228245; doi:10.1128/JCM.01757-16)
Supplement: Supplemental material [file JCM.01757-16_zjm999095327s1.pdf]

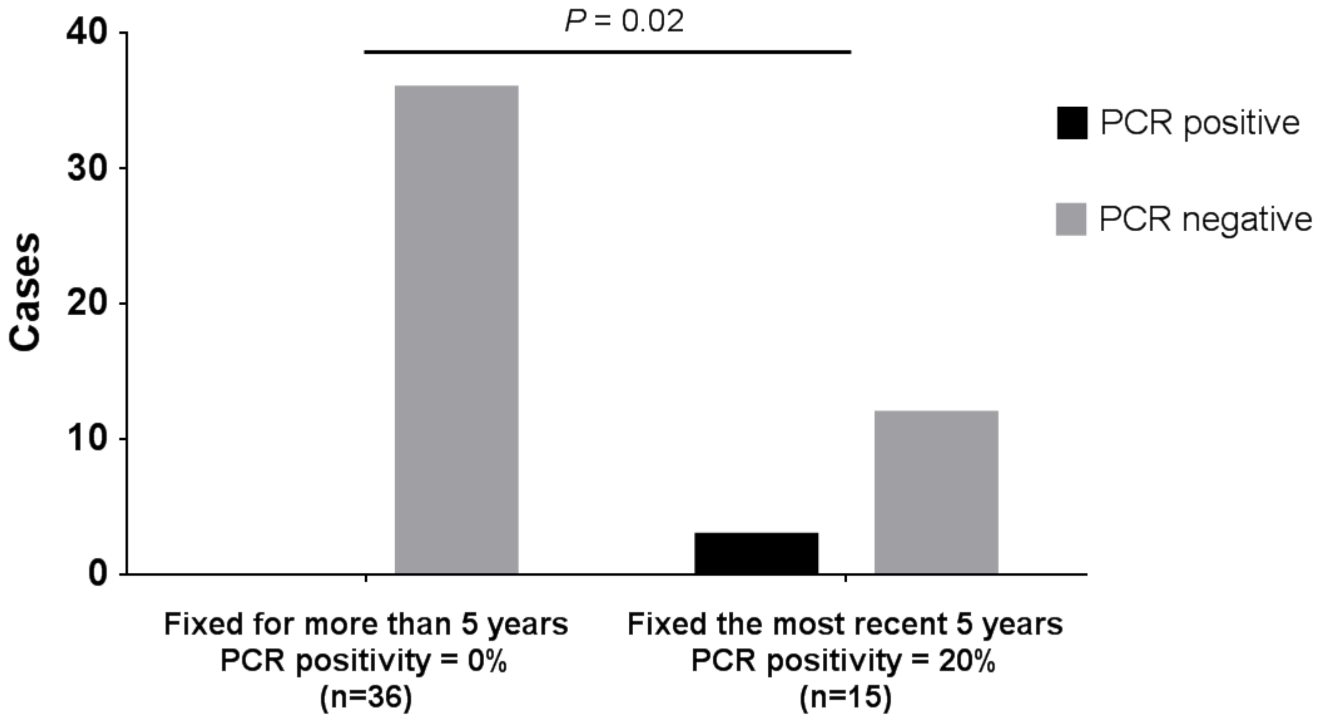

Supplementary Figure 1. Results of polymerase chain reaction (PCR) using histologically-negative samples (n=51). The PCR-positivity rate of tissue samples fixed for more than 5 years was significantly lower than the rate of samples fixed in the most recent 5 years (Fisher's exact test, *P*=0.02).
